# Draft Genome Sequence of the Basidiomycetous Fungus *Flammulina velutipes* TR19

**DOI:** 10.1128/genomeA.00505-16

**Published:** 2016-06-09

**Authors:** Atsushi Kurata, Yasuhisa Fukuta, Miho Mori, Noriaki Kishimoto, Norifumi Shirasaka

**Affiliations:** Faculty of Agriculture, Kindai University, Nakamachi, Nara City, Nara, Japan

## Abstract

Here, we report the draft genome sequence of *Flammulina velutipes* TR19, which was newly isolated from commercial strains in Japan. The genes related to fruiting body formation in the basidiomycete were identified by whole-genome analysis.

## GENOME ANNOUNCEMENT

Fruiting bodies of basidiomycetous fungi are referred to as mushrooms. The production of mushrooms is important in the food, pharmaceutical, and biotechnological industries (1). Although the demand for mushrooms has expanded all over the world, it is difficult to constitutively develop fruiting bodies in many basidiomycetes (2). Fruiting body formation is proposed to start with the formation of an aggregate from mycelium, the formation of a primordium, and the outgrowth of the mature fruiting body (3). Although insights into fruiting body development have been provided in several basidiomycetes (4–6), the complex molecular mechanism in each developmental stage remains to be revealed.

We newly isolated *Flammulina velutipes* TR19 by mating. *F. velutipes*, which belongs to the order *Agaricales*, is known as winter mushroom. Strain TR19 stably forms a fruiting body using potato dextrose agar (PDA) liquid medium. In order to elucidate the molecular mechanisms in formation of fruiting body, we sequenced the whole genome of strain TR19 using the Illumina HiSeq (Hokkaido System Science Co., Ltd., Japan). The genomic DNA of dikaryotic TR19 cells was prepared using Isoplant II (Nippon Gene Co., Ltd., Japan). Paired-end libraries of 100-bp fragments were prepared, and the genome sequencing generated 45,722,056 raw reads covering a total of 4,618 Mbp (Phred >Q30, 92.52%). Quality-trimmed DNA reads conferring 132-fold coverage were assembled *de novo* by Velvet 1.2.08. As a result, the genome sequence comprised 5,130 contigs totaling 34,792,959 bp, with an average length of 6,782 bp (largest, 792,880 bp; smallest, 169 bp). An *N*_50_ contig length of 150,115 bp and a *N*_90_ contig length of 8,389 bp were obtained. The G+C content was 49.6%. All assembly data were deposited in the DDBJ/EMBL/GenBank nucleotide sequence database.

The gene prediction and annotation were carried out using an Augustus tool (7) and the Microbial Genome Annotation Pipeline (MiGAP, http://www.migap.org) (8, 9). A total of 13,843 protein-coding genes were predicted. As a result, we identified six genes encoding proteins involved in the fruiting body development by MiGAP and local blast program with Geneious R9 (Biomatters Ltd., Auckland, New Zealand), namely, two hydrophobin genes (*hyd1* and *hyd2*), one mitochondrial ATP-synthase gene (*atp*), one fruiting body-specific gene (*fds*), and two transcription factor genes (*fst* and *gat*). The nucleotide sequences of *hyd1*, *hyd2*, and *atp* from strain TR19 were similar to those of the hydrophobin gene *fvh1* (GenBank accession no. AB026721.1) from *F. velutipes*, the hydrophobin gene *Fv-hyd1* (GenBank accession no. AB126686.1) from *F. velutipes*, and the mitochondrial ATP-synthase gene *atp*9 (GenBank accession no. NC_021373.1 [83400.83621]) from *F. velutipes*, respectively, with 100% identities. The nucleotide sequences of *fds*, *fst*, and *gat* from strain TR19 exhibited the closest similarities to those of the fruiting body-specific gene *fds* (GenBank accession no. D83658.1) from *F. velutipes* with 81% identity*,* the transcription factor gene *fst3* (GenBank accession no. XM_003031274) from *Schizophyllum commune* with 80% identity (10), and the transcription factor gene *gat1* (GenBank accession no. XM_003036543) from *S. commune* with 71% identity (10), respectively. We plan to analyze the functions of the genes and detect other genes involved in fruiting body development in the genome of *F. velutipes* TR19.

### Nucleotide sequence accession number.

The genome sequence of *F. velutipes* TR19 is available in DDBJ/EMBL/GenBank under the accession no. BDAN00000000.
